# COVID-19 Therapeutics for Low- and Middle-Income Countries: A Review of Candidate Agents with Potential for Near-Term Use and Impact

**DOI:** 10.4269/ajtmh.21-0200

**Published:** 2021-07-16

**Authors:** Daniel Maxwell, Kelly C. Sanders, Oliver Sabot, Ahmad Hachem, Alejandro Llanos-Cuentas, Ally Olotu, Roly Gosling, James B. Cutrell, Michelle S. Hsiang

**Affiliations:** 1 Department of Medicine, University of Texas Southwestern Medical Center, Dallas, Texas;; 2 Pandemic Response Initiative, Institute for Global Health Sciences, University of California, San Francisco, San Francisco, California;; 3 Department of Pediatrics, Stanford University, Stanford, California;; 4 Institute of Tropical Medicine Alexander von Humbolt, Universidad Peruana Cayetano Heredia, Lima, Peru;; 5 Clinical Trials and Interventions Unit, Ifakara Health Institute, Bagamoyo, Tanzania;; 6 Department of Pediatrics, University of Texas Southwestern Medical Center, Dallas, Texas;; 7 Department of Pediatrics, University of California, San Francisco, San Francisco, California

## Abstract

Low- and middle-income countries (LMICs) face significant challenges in the control of COVID-19, given limited resources, especially for inpatient care. In a parallel effort to that for vaccines, the identification of therapeutics that have near-term potential to be available and easily administered is critical. Using the United States (US), European Union (EU), and World Health Organization (WHO) clinical trial registries, we reviewed COVID-19 therapeutic agents currently under investigation. The search was limited to oral or potentially oral agents, with at least a putative anti-SARS-CoV-2 virus mechanism and with at least five registered trials. The search yielded 1,001, 203, and 1,128 trials, in the US, EU, and WHO trial registers, respectively. These trials covered 13 oral or potentially oral repurposed agents that are currently used as antimicrobials and immunomodulatory therapeutics with established safety profiles. The available evidence regarding proposed mechanisms of action, potential limitations, and trial status is summarized. The results of the search demonstrate few published studies of high quality, a low proportion of trials completed, and the vast majority with negative results. These findings reflect limited investment in COVID-19 therapeutics development compared with vaccines. We also identified the need for better coordination of trials of accessible agents and their combinations in LMICs. To prevent COVID-19 from becoming a neglected tropical disease, there is a critical need for rapid and coordinated efforts in the evaluation and deployment of those agents found to be efficacious.

## INTRODUCTION

The coronavirus disease 2019 (COVID-19) pandemic is causing devastating long-term health and socioeconomic impact in low- and middle-income countries (LMICs).^1^ Although lower population density and younger age demographics in some LMICs may be protective against high COVID-19 mortality,^2^ poor health infrastructure, including limited numbers of hospital beds, health care providers, and basic supplies, can lead to overwhelmed local health systems with relatively few cases.^3^ Although vaccine research and deployment have moved forward at an unprecedented pace,^4^^,^^5^ therapeutic development has not received the same level of political and financial commitment. There are several reasons for renewed focus and investment in therapeutics. Widespread vaccination coverage for LMICs will take years due to financial and operational barriers.^6^^–^^8^ Even with widespread coverage, treatments for those who cannot or do not receive the vaccine will still be needed. Waning immunity and potential emergence of vaccine resistance among new variants may also compromise impact of vaccines. As has been the case with other vaccine-preventable illnesses such as bacterial meningitis, pertussis, and influenza, therapeutics can play an important role in disease control alongside vaccines.

To date, the only available therapies with known efficacy in COVID-19 are dexamethasone,^9^ remdesivir,^10^ and tocilizumab^11^ for moderate and severe disease. There is an urgent need for therapeutics that act early in disease to prevent progression and work as pre- or post-exposure prophylaxis to prevent infection and stem transmission. Monoclonal antibodies are the only therapy with known efficacy for mild disease,^12^ but as injectable agents, they are impractical at home or an outpatient clinic, particularly for rural populations that live far from health centers. Oral, transmucosal, or transdermal agents will be preferable, followed by intranasal or inhaled formulations through a metered-dose inhaler, and then nebulized formulations. An additional consideration is cost, with low cost being ideal due to limited governmental health care budgets and a large proportion of the population paying out-of-pocket for care in LMICs.

Since the start of the pandemic, hundreds of available chemicals and drugs with antiviral properties that could be effective as COVID-19 therapeutics have been screened and studied. Here we review candidates that are currently in advanced clinical trials with the potential to be rapidly available. To facilitate widespread use in the outpatient and community settings, we limited the search to agents for which administration is oral or nasal. Recognizing that findings have relevance to all settings, our study places special emphasis on relevance to lower resource settings by excluding drugs that have higher resource requirements for administration.

## METHODS

Using the term “COVID-19” and its synonyms (“COVID,” “SARS-CoV-2,” “severe acute respiratory syndrome coronavirus 2,” “2019-nCoV,” “2019 novel coronavirus,” “Wuhan coronavirus”), we searched for trials registered in the US National Library of Medicine (clinicaltrials.gov), EU Clinical Trials Register (clinicaltrialsregister.eu), and the World Health Organization (WHO) International Clinical Trials Registry Platform (WHO ICTRP). On March 29, 2021, a search was conducted and limited to trials that were interventional, randomized and with a trial intervention classified as “drug.” In a previous search, we included drugs with at least three registered trials, but this led to a high proportion of the drugs with only pending or negative results. To maximize the possibility of a sufficient evidence base on which to make recommendations, either now or in the near future, drugs that were studied in at least five trials were included in the final review. Drugs were excluded if they had no putative antiviral mechanism or if there were no oral or intranasal options for administration. On April 23, 2021, the list of drugs meeting eligibility criteria was then used to identify and include all trials registered in the US, EU, and WHO ICTRP registries. Data regarding the number and status of trials were collected and summarized. PubMed was also searched using the drug name and COVID-19, with results screened for randomized controlled trials (RCTs) of the drug in question. Data regarding therapeutic class, examples of current uses (for repurposed drugs), mechanism of action, and potential cost considerations were summarized, as were trial data regarding samples size, primary endpoint, and overall result.

## RESULTS

**Figure 1.**
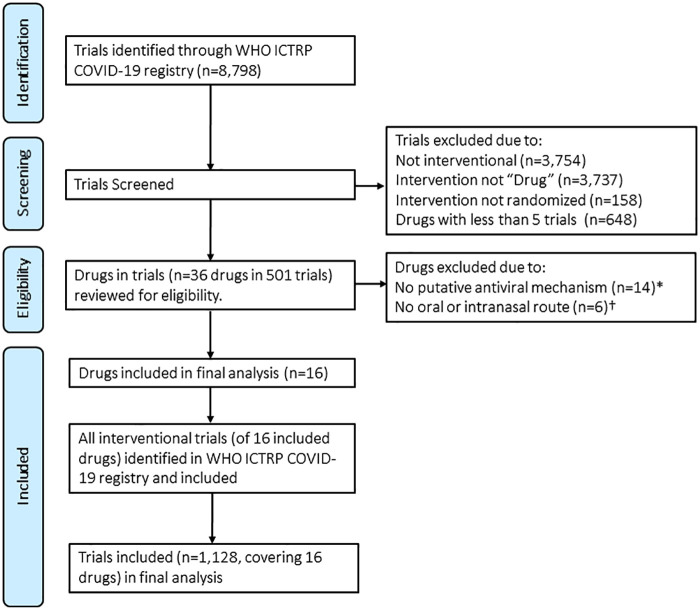
Flow diagram for drug selection based on results of trial search in World Health Organization International Clinical Trials Registry Platform (WHO ICTRP). *Drugs without putative antiviral mechanism (*N* = 14): anakinra, cholecalciferol, clazakizumab, dexamethasone, mavrilimumab, melatonin, methylprednisolone, nitric oxide, povidone-iodine (as no appreciable intracellular antiviral activity anticipated for studied intervention), prednisone, rivaroxaban, sargramostim, sarilumab, tocilizumab. †No oral or intranasal options for administration (*N* = 6): convalescent plasma, enoxaparin, LY3819253, nafamostat, REGN10933, remdesivir. ‡This search included all interventional trials and was not limited to trials where the intervention was listed as “Drug” or “randomized”. This figure appears in color at www.ajtmh.org.

The results of the search are shown in Table 1. The search yielded 1,001, 203, and 1,128 trials, in the US, EU, and WHO trial registers, respectively. Because the WHO ICTRP is a combination of the US, EU, and other registers such as the Chinese Clinical Trial Registry, we present a Preferred Reporting Items for Systematic Reviews and Meta-Analyses (PRISMA) flow diagram for the WHO ICTRP search (Figure 1). The ICTRP trials covered 36 unique drugs, of which 14 were excluded for having no putative antiviral mechanism and six were excluded for not having an oral or intranasal route of administration. There were 16 agents, of which three were grouped with another structurally similar agent. The final list of 13 agents included eight antimicrobials (four antiparasitics, one antibiotic, and four antivirals) and four immunomodulatory therapeutics, all repurposed agents with established safety profiles. All were oral except interferon (IFN), for which nasal routes of administration are being evaluated. The agents with > 100 trials registered included azithromycin, hydroxychloroquine (HCQ), IFN, and lopinavir/ritonavir. Data regarding therapeutic class, examples of current uses, mechanism of action, potential limitations, and number and status of trials are shown in Table 1 and summarized in the following sections. Only 197 (19.7%), 6 (3%), and 95 (8.4%) trials in the U.S., EU, and WHO registries, respectively, were completed or in Phase 4. Available efficacy data are described in the following sections and summarized in Table 2. Information regarding five agents (bemcentinib, doxycycline, fingolimod and opaganib, maraviroc, and umifenovir) that did not meet the final search criteria but were identified in a prior search are shown in the Supplemental Appendix.

**Table 1:** COVID-19 candidate therapeutics with highest potential to be available and easily administrable in the near future

Candidate therapeutic	Therapeutic class	Examples of current uses	Rationale/mechanism of action	Limitations/potential obstacles	Trials in U.S. National Library of Medicine		Trials in European Union Clinical Trials Register		Trials in WHO International Clinical Trials Registry Platform	
					Listed	Completed	Listed	Completed	Listed	Phase 4
Azithromycin	Macrolide antibiotic	Community-acquired pneumonia	Alkalinization of endosomal vesicles/lysozomes, potentially inhibiting endocytosis and/or uncoating of enveloped viruses	No RCTs completed to date except with HCQ/CQ, where it was not shown to be helpful and some signal for harm	126	28	27	0	115	4
Baricitinib	JAK1/JAK2 inhibitor	Rheumatoid arthritis	May inhibit viral entry via clathrin-dependent endocytosis; immunomodulator	May have cost and availability issues as IP owned by Eli Lilly	18	2	11	1	16	2
Camostat	Serine protease inhibition	Chronic pancreatitis	TMPRSS2 inhibitor	Trials pending	25	1	6	0	26	1
CQ	Antimalarial	Malaria, autoimmune conditions, rheumatologic diseases	Block viral entry (see HCQ); immunomodulator	More toxicity than HCQ with less preclinical evidence of antiviral activity; negative clinical trial	91	20	11	0	49	13
HCQ	Antimalarial	Malaria, autoimmune conditions, rheumatologic diseases	Block viral entry by impairing terminal glycosylation of ACE2 receptors, proteolytic processing, and endosomal acidification; immunomodulator	Multiple negative trials in all phases of illness, from pre-exposure prophylaxis to treatment of hospitalized patients	274	64	70	2	382	33
Colchicine	Antiinflam.	Gout, Behcet disease	Reduce neutrophil chemotaxis, cytokine production, IL-1beta	Narrow therapeutic index	32	5	8	1	46	0
Favipiravir	Antiviral	Investigations for Nipah virus, Ebola, and other flaviviruses	RNA-dependent RNA polymerase inhibition	Safety concerns regarding increases in blood uric acid and teratogenic potential	52	13	9	0	67	3
IFN	Biologic response modifier	Hepatitis C, multiple sclerosis	Strengthens IFN response to SARS-CoV-2 infection	Subcutaneous administration (though nasal administration possible); large negative trial	147	12	20	0	99	10
Ivermectin	Antiparasitic	Onchocerciasis, lymphatic filariasis, soil-transmitted helminths, scabies	Nuclear transport inhibition	May be difficult to achieve therapeutic dose	65	21	6	1	91	6
Lopinavir/ ritonavir	Antiviral	HIV (older regimen)	Protease inhibition	Large negative trial	92	18	21	0	136	15
Molnupiravir (formerly MK-4482 and EIDD-2801)	Antiviral	Developed for influenza, investigated for SARS, MERS	Nucleoside analogue	Early-phase trials; may have cost and availability issues as IP owned by Ridgeback; possible mutagenicity (disputed)	6	2	0	0	10	0
Niclosamide	Antiparasitic	Tapeworm infections	Inhibits viral entry by change in endosomal pH; inhibit autophagy	Trials few in number and in early stages	13	0	2	0	12	2
Nitazoxanide	Antiparasitic	Cryptosporidiosis, broad-spectrum antiparasitic	Phosphorylation of protein kinase activated by dsRNA; immunomodulator	Trials in early stages	26	6	0	0	28	4
Ruxolitinib	JAK1/2 kinase inhibitor	Myelofibrosis, polycythemia vera, acute graft-versus-host disease	Potentially inhibits AAK1, and prevents clathrin-mediated viral endocytosis; antiinflam. agent for cytokine storm	Not as potent AAK1 inhibitor as baricitinib	16	1	9	1	24	0
Sofosbuvir with daclatasvir or ledipasvir	Antiviral	Hepatitis C	RNA polymerase inhibition	Concerns regarding partial treatment of undiagnosed hepatitis C virus	12	3	0	0	22	2
Tofacitinib	JAK1/2 kinase inhibitor	Autoimmune diseases including rheumatoid arthritis, psoriatic arthritis, and ulcerative colitis	Antiinflam. agent for cytokine storm	No detectable inhibition of AAK1	6	1	3	0	5	0
Totals					1,001	197	203	6	1,128	95

Antiinflam. = antiinflammatory; CQ, chloroquine; HCQ, hydroxychloroquine; IFN = Interferon; IL = interleukin; IP = intellectual property; MERS = Middle East respiratory syndrome; N/A = not available; PCR = polymerase chain reaction; RCT = randomized controlled trial; SARS = severe acute respiratory syndrome.

**Table 2:** Published RCTs for potential COVID-19 drugs

Drug	First author	Journal	Assigned to treatment (*n*)	Primary endpoint(s)	Result
Azithromycin	PRINCIPLE Group	*Lancet*	540	Time to recovery, hospital admission or death	Neg
Azithromycin	RECOVERY Group	*Lancet*	7,763	28 day all-cause mortality	Neg
Azithromycin	Furtado	*Lancet*	214	Clinical status at day 15	Neg
Azithromycin	Sekhavati	*Int J Antimicrob Agents*	56	Multiple endpoints	Neg
Azithromycin	Cavalcatani	*N Engl J Med*	217	Clinical status at 15 days	Neg
Baricitinib	Kalil	*N Engl J Med*	515	Recovery time	Pos
Camostat	N/A	N/A	N/A	N/A	N/A (no RCTs)
CQ	Thakar	*Indian J Med Res*	30	Multiple endpoints	Neg
HCQ	Barnabas	*Ann Intern Med*	407	Preventing infection with SARS-CoV-2	Neg
HCQ	Solidarity Consortium	*N Engl J Med*	954	Mortality, ventilation, length of hospitalization	Neg
HCQ	Chen	*PLoS One*	21	Shortening viral shedding	Neg
HCQ	Self	*JAMA*	242	Clinical status 14 days after randomization	Neg
HCQ	Lyngbakken	*Nat Commun*	27	Decline in oropharyngeal viral load	Neg
HCQ	RECOVERY Group	*N Engl J Med*	1,561	Mortality, ventilation, length of hospitalization	Neg
HCQ	Abella	*JAMA Intern Med*	66	Incidence of SARS-CoV-infection during Ppx	Neg
HCQ	Abd-Elsalam	*Am J Trop Med Hyg*	97	Recovery within 28 days, ventilation, death	Neg
HCQ	Cavalcatani	*N Engl J Med*	221	Clinical status at 15 days	Neg
HCQ	Skipper	*Ann Intern Med*	244	Reducing symptom severity in early mild COVID-19	Neg
HCQ	Tang	*BMJ*	75	Negative conversion by day 28	Neg
Colchicine	Mareev	*Kardiologiia*	26 (approx)	Change in SHOKS-COVID clinical condition score	Pos
Colchicine	Isabel Lopes	*RMD Open*	38	Need for supplemental oxygen, length of stay	Pos
Colchicine	Deftereos	*JAMA Netw Open*	55	Time to clinical deterioration	Pos
Favipiravir	Dabbous	*Arch Virol*	48	No primary endpoint defined	Neg
Favipiravir	Zhao	*Biomed Pharacother*	21	Lung lesion remission	Neg
Favipiravir	Udwadia	*Int J Inf Dis*	75	Cessation of viral shedding	Neg
Favipiravir	Khamis	*Int J Inf Dis*	44	Biomarkers, length of stay, discharge, 14-day mortality	Neg
Favipiravir	Lou	*Eur J Pharm Sci*	10	Viral clearance, time to clinical improvement	Neg
IFN	Jagannathan	*Nat Commun*	60	Shorten duration of viral shedding	Neg
IFN	Li	*Ann Med*	46	Time to clinical improvement	Neg
IFN	Solidarity Consortium	*N Engl J Med*	2,063	Mortality, ventilation, length of hospitalization	Neg
IFN	Monk	*Lancet Respir Med*	48	Change in WHO Ordinal Scale	Pos
IFN	Rahmani	*Int Immunopharmacol*	40	Time to clinical improvement	Pos
IFN	Zheng	*Int J Inf Dis*	60	Viral clearance on treatment day 6	Pos
IFN	Davoudi-Monfared	*Antimicrob Agents Chem*	46	Time to clinical response	Neg
IFN	Hung	*Lancet*	86	Time to negative nasopharyngeal PCR	Pos
Ivermectin	Ahmed	*Int J Inf Dis*	24	Days to virologic clearance	Pos
Ivermectin	Mahmud	*J Int Medical Res*	400	Time to clinical recovery	Pos
Ivermectin	Lopez -Medina	*JAMA*	400	Time to resolution	Neg
LPV/r	Solidarity Consortium	*N Engl J Med*	1,411	Mortality, ventilation, length of hospitalization	Neg
LPV/r	Cao	*N Engl J Med*	199	Time to clinical improvement	Neg
LPV/r	Recovery Group	*Lancet*	1,616	Mortality, ventilation, length of hospitalization	Neg
Molnupiravir	N/A	N/A	N/A	N/A	N/A (no RCTs)
Niclosamide	N/A	N/A	N/A	N/A	N/A (no RCTs)
Nitazoxanide	N/A	N/A	N/A	N/A	N/A (no RCTs)
Ruxolitinib	Cao	*J Allergy Clin Immunol*	22	Time to clinical improvement	Neg
Sofosbuvir	Khalil	*Acta Biomed*	42	5 primary endpoints; 4 negative	Neg
Sofosbuvir	Roozbeh	*J Antimicrob Chemother*	27	Symptom alleviation at 7 days	Neg
Sofosbuvir	Eslami	*J Antimicrob Chemother*	35	Time to discharge	Pos
Sofosbuvir	Sadeghi	*J Antimicrob Chemother*	33	Clinical recovery within 14 days of treatment	Pos
Sofosbuvir	Abbaspour	*J Antimicrob Chemother*	24	Length of hospital stay	Neg
Tofacitinib	N/A	N/A	N/A	N/A	N/A (no RCTs)

CQ = chloroquine; HCQ = hydroxychloroquine; IFN = Interferon; N/A = not available; PCR = polymerase chain reaction; RCT = randomized controlled trial; WHO = World Health Organization.

### Azithromycin.

This macrolide antibiotic has been demonstrated to have a high selectivity index (SI), based on half-maximal inhibitory concentration (IC50) divided by maximal cytotoxic concentration (CC50).^13^ However, in large RCTs, it has been combined with HCQ or chloroquine (CQ), and this combination has not shown to be of benefit with potential signals of harm.^14^^–^^17^ Although no high-quality RCTs of azithromycin alone are available yet, a trial of mass administration to children in Niger resulted in 8- to 14-fold reductions in various coronaviruses based on polymerase chain reaction (PCR) of the nasopharynx.^18^ Further results from at least one additional large RCT are expected.^19^

### Baricitinib, Ruxolitinib, Tofacitinib: Janus kinase/signal transducer and activator of transcription inhibitors.

In an attempt to mitigate the hyperinflammatory cytokine storm associated with COVID-19, janus kinase/signal transducer and activator of transcription (JAK/STAT) inhibitors baricitinib, ruxolitinib, and tofacitinib have been investigated as potential therapeutics. These agents inhibit the intracellular pathways of cytokines known to be elevated in severe COVID-19. As an AP2 Associated Kinase 1 (AAK1) inhibitors, baricitinib and ruxolitinib may also block viral entry and intracellular assembly of virus particles.^20^^,^^21^ Several cases series have demonstrated a beneficial effect of baricitinib in treatment of COVID-19 patients.^22^^,^^23^ The ACTT-2 trial (Adaptive COVID-19 Treatment Trial—2) was a multinational, multicenter placebo-controlled double randomized trial that demonstrated the combination therapy of remdesivir and baricitinib versus remdesivir alone lowered hospital length of stay (8 versus 7 days, *P* = 0.04), and there was a trend toward lower mortality at 29 days (5.1 versus 7.8%, *P* = 0.09).^24^ In a small randomized trial, patients treated with ruxolitinib did not have statistically significant reductions in time to clinical improvement or survival.^25^ An unpublished Phase III multicenter, randomized, double blind placebo-controlled trial comparing ruxolitinib with standard of care failed to meet its primary endpoint of severe complications.^26^ Several trials of tofacitinib for severe COVID-19 are pending recruitment; one has been completed but results are not published yet.

### Camostat.

Camostat and the closely related nafamostat are serine protease inhibitors with decades of clinical use in disseminated intravascular coagulation and pancreatitis.^27^ Unlike other agents for SARS-CoV-2, they act on a human target, rather than a viral target, called transmembrane serine protease 2 (TMPRSS2).^28^ Because this is a necessary agent in the viral spike protein’s function, these agents can partially inhibit SARS-CoV-2 entry into lung epithelial cells. Although both agents show promising in vitro data, nafamostat inhibited SARS-CoV-2 S-mediated entry into host cells with approximately 15-fold higher efficiency than camostat at a low EC50.^29^ However, due to the requirement for intravenous administration, nafamostat has been overshadowed by oral camostat in ongoing clinical trials.

### Colchicine.

Colchicine is an inexpensive antiinflammatory oral medication used for gout, familial Mediterranean fever, pseudogout, sarcoid, pericarditis, and psoriatic arthritis. As an inhibitor of microtubule assembly, cochicine may inhibit coronavirus replication, as has been shown with Zika and dengue virus.^30^^,^^31^ Colchicine may also inhibit viral entry^32^ and NLPR3 inflammasome activation, which is thought to mediate lung inflammation in COVID-19.^33^^,^^34^ We identified 17 clinical trials of colchicine for COVID-19 infection in both the inpatient and outpatient settings. The ColCORONA trial was a randomized, double-blind, placebo-controlled trial that was halted early due to variable recruitment rates and promising early results. The primary outcome of prevention of hospitalization and death at 30 days was not different between colchicine and placebo. In the subset of PCR-positive patients, colchicine was superior to placebo in preventing hospitalization and death at 30 days with a number needed to treat to prevent one hospitalization was 72.^35^ The colchicine arm in the UK RECOVERY trial, a large adaptive RCT, was halted due to lack of efficacy compared with standard of care in preliminary analyses of the 28-day mortality outcome among > 11,000 randomized patients.^36^

### Favipiravir.

Favipiravir is a nucleoside analogue shown to have in vitro activity against SARS-CoV-2 in Vero cells.^37^ Despite its less favorable SI, favipiravir was demonstrated to be 100% effective in a post-exposure prophylaxis mouse model after challenge with aerosolized Ebola virus, a virus that has a similar IC50.^38^^,^^39^ Clinical data on favipiravir for SARS-CoV-2 are still limited. A trial of 80 patients compared favipiravir with IFN-alpha against lopinavir/ritonavir with interferon alpha. Although the favipiravir group had shorter time to viral clearance (4 versus 11 days), more frequent improvement in chest imaging (91% versus 62%), and fewer adverse events (11% versus 55%), these results are limited by lack of blinding and randomization.^37^ An open-label, multicenter phase 3 clinical trial randomized 150 patients with mild to moderate COVID-19 to favipiravir versus standard of care, and preliminary results showed shorter time to clinical cure and viral clearance.^40^

### Hydroxychloroquine.

Many trials have been undertaken to investigate the antimalarial and rheumatologic therapeutic CQ and its derivative HCQ for treatment of COVID-19, but results thus far have been largely negative. Here we focused on HCQ, which is less toxic and more active against SARS-CoV-2 in vitro than CQ.^41^ The SOLIDARITY trial of the WHO has reported interim results indicating that none of the four treatments tested, including HCQ, reduced 28-day mortality, initiation of ventilation, or duration of hospitalization compared with usual care.^42^ The RECOVERY Collaborative found no difference in 28-day mortality in 1,561 hospitalized patients randomized to HCQ or usual care.^43^ A trial of early treatment randomizing 491 patients also found no difference in symptoms.^44^ For prophylaxis, dozens of other trials are underway. One trial of post-exposure prophylaxis in community members and health workers found it to be ineffective, although there were limitations of this pragmatic trial.^45^ Another randomized double-blind, placebo-controlled trial among health care workers showed no difference between post-exposure prophylaxis of HCQ (600 mg daily for 8 weeks) versus placebo.^46^ Another randomized trial of 1,483 health care workers showing no reduction in incidence of COVID-19 using once or twice weekly HCQ pre-exposure prophylaxis.^47^ In summary, all phases of illness have been studied, and HCQ has not been found to be effective to date.

### IFN, particularly beta-1a and beta-1b.

As cytokine mediators that trigger the cellular immune response to viral infections, IFNs have been used and studied as antiviral therapy for several viral infections.^48^ SARS-CoV-2 has been shown to induce particularly low levels of IFN-1.^49^ In vitro, high concentrations of IFN beta-1a completely protected Vero E6 cells without evidence of cytotoxicity in uninfected cells.^50^ IFN beta-1a has been evaluated in an open-label, randomized trial of 127 patients comparing lopinavir/ritonavir (LPV/r) to triple therapy with LPV/r, IFN beta-1a, and ribavirin.^51^ Triple therapy, when administered in the first 7 days, was superior in symptom relief, shortening duration of viral shedding, and shortening duration of hospital stay, all with no difference in adverse events.^51^ Subgroup analysis of the trial suggested that IFN beta-1b was the major driver of clinical differences between groups. More recently, an Iranian trial of 81 patients comparing the national protocol of HCQ plus LPV/r or atazanavir-ritonavir versus the same protocol plus subcutaneous IFN beta-1a showed no change in time to clinical response (the primary outcome) but did demonstrate decreased 28-day mortality in the IFN beta-1a group (19% versus 43%, *P* = 0.015).^52^ However, the WHO SOLIDARITY trial did not show any benefit in mortality, progression to ventilation, or duration of hospitalization.^42^ Although IFN is typically given as an intravenous or subcutaneous drug, several trials in the treatment of COVID-19 are evaluating other IFN formulations including inhaled, nebulized, nasal spray, and nasal drops.^53^^–^^56^

### Ivermectin.

Given its wide safety margin, ivermectin has been used broadly in mass distribution campaigns to treat illnesses such as onchocerciasis and lymphatic filariasis. More recently, it has been found to limit infection by dengue virus, West Nile Virus, and influenza, with antiviral activity attributed to inhibition of RNA virus interactions with host nuclear transport proteins.^57^ Ivermectin has been tested in vitro against SARS-CoV-2, demonstrating reduction in viral RNA in infected cells.^57^ However, concentrations needed to achieve viral activity appear to be much higher than what standard doses can achieve in serum. Nonetheless, the drug can concentrate to higher levels in lung.^58^^,^^59^ An early trial demonstrated a 5-day course of ivermectin in adult patients with mild COVID-19 was safe and decreased time to virological clearance compared with placebo (9.7 versus 12.7 days, *P* = 0.02).^60^ However, a subsequent, larger RCT in 476 patients with mild COVID-19 failed to show any difference in time to resolution of symptoms with a 5-day course of ivermectin compared with placebo.^61^ In a trial of 400 patients, those given ivermectin with doxycycline versus placebo recovered earlier, were less likely to progress to more serious disease, and were more likely to be COVID-19 negative by reverse transcriptase PCR on day 14.^62^ Larger, randomized, and blinded trials are needed to fully evaluate its role in COVID-19 management. Lastly, ivermectin’s benefit in COVID-19 treatment could be attributed to preventing *Strongyloides* hyperinfection in patients treated with dexamethasone.^63^ Because *Strongyloides stercoralis* is hyperendemic in many LMICs, this could significantly reduce morbidity.

### Lopinavir/ritonavir.

Lopinavir is a well-studied protease inhibitor used to treat HIV type 1. It is administered in combination with ritonavir, a cytochrome P450 inhibitor, to increase its plasma half-life. Because lopinavir/ritonavir (LPV/r) was previously identified as having in vitro activity against SARS-CoV, this agent was used in an early, open-label clinical trial in a Wuhan hospital (*N* = 199), with participants randomized to LPV/r or standard of care.^64^ Although there was a trend toward lower mortality (19.2% versus 25%), it was not statistically significant. It should be noted that three of the 19 patients who died in the experimental arm died less than 24 hours after randomization and never received LPV/r. However, the RECOVERY collaborative found in a large, multicenter randomized trial that this treatment was not associated with reduction in 28-day mortality, duration of hospitalization, or progression to ventilation or death.^65^

### Molnupiravir (formerly MK-4482 and EIDD-2801).

Molnupiravir is an orally bioavailable prodrug of beta-D-N4-hydroxycyctidine that has demonstrated antiviral activity against Venezuelan equine encephalitis virus.^66^ More recently, it has shown in vitro activity against SARS-CoV-2^67^ and a favorable SI.^67^ Oral molnupiravir improved lung function in mice infected with Middle East respiratory syndrome (MERS-CoV). Currently, it is in phase 2/3 trials in inpatient and outpatient settings.^68^ The inpatient portion of the trial, MOVe-IN, was discontinued due to clinical futility, with only the phase 3 of the MOVe-OUT study for outpatients currently planned to start enrolling in spring 2021. In the dose-determining phase of MoVe-OUT, investigators found that the percentage of patients who died or were hospitalized decreased, but the study did not have sufficient power to establish a clinical benefit.^69^

### Niclosamide.

Niclosamide is another antiparasitic with a well-established safety profile that is currently on the WHO Model List of Essential Medicines.^70^ Although it is used primarily for treatment of cestode infections, niclosamide has been found to have a low IC50 against SARS-CoV-2. Possible mechanisms of action include blocking endocytosis, inhibition of viral replication, and inhibiting receptor-mediated endocytosis.^71^ Despite its potential, reported clinical trials of this agent are limited.

### Nitazoxanide.

Although not on the WHO List of Essential Medicines, the antiparasitic nitazoxanide has a long history of safety and tolerability since its discovery 30 years ago. It was investigated as an antiviral against MERS in 2016,^72^ as well as against influenza, with variable results.^73^^,^^74^ Against SARS-CoV-2, nitazoxanide has a promising ratio of plasma concentration to IC50 for SARS-CoV-2 of 14:1 with standard dosing and could be produced at a low daily cost of $0.10.^75^ As of January 14, 2021, 24 clinical trials of nitazoxanide are listed on clinicaltrials.gov, but only three have been completed. Notably, in the Brazilian SARITA-2 trial, it showed no difference in symptoms at 5 days but did decrease viral load without serious adverse events noted.^76^

### Sofosbuvir/Daclatasvir and Sofosbuvir/Ledipasvir.

The combination of sofosbuvir and either daclatasvir or ledipasvir has been used to treat hepatitis C and has proven effective and well tolerated for that indication.^77^ More recently, in silico studies have been carried out identifying both sofosbuvir and daclatasvir as potential inhibitors of SARS-CoV-2.^78^^–^^80^ This combination offer well-established safety and tolerability as combination therapy and has been shown to be safe in patients with renal impairment.^81^ Three small trials, sometimes in combination with ribavirin, have been published. All three demonstrated a trend toward reduced mortality in the treatment group, with one achieving statistical significance (*P* = 0.02),^82^^–^^84^ but numbers were too small to draw definitive conclusions about efficacy.

## DISCUSSION

We identified 13 candidate COVID-19 therapeutics that are easily administrable, currently being evaluated in multiple clinical trials and have potential for near-term use and impact in LMICs. All identified agents were repurposed and are currently being used as antimicrobials and immunomodulatory therapeutics. Repurposed agents have established safety and higher likelihood of avoiding licensing costs compared with novel agents. If these drugs are found to be efficacious, these characteristics could facilitate rapid and widespread access in LMICs, particularly for agents that are already widely used in LMICs such as azithromycin and certain antiparasitics. However, despite the high number of total trials, few published studies were of high quality, study endpoints were highly variable, and only a small proportion of trials have been completed. Although some agents, including baricitinib, colchicine, interferon, and sofosbuvir, have demonstrated some potential utility, the vast majority of results have been negative. There are also a disproportionate number of trials for unproven therapies including azithromycin, HCQ, IFN, ivermectin, and lopinavir/ritonavir. Taken together, these findings reflect limited investment and a lack of coordination in COVID-19 therapeutics development compared with vaccines.

A potential limitation of this work is that the landscape for the development of COVID-19 therapeutics is rapidly changing, and our review may not have captured all agents with high potential for near-term potential for use and impact in LMICs. However, this review provides a framework and reference on which COVID-19 drug development for LMICs can be tracked moving forward.

It should be noted that the therapeutics arm of the Access to COVID-19 Tools Accelerator, with funding from the Bill and Melinda Gates Foundation, the Wellcome Trust, the Mastercard Impact Fund, National Institutes of Health, and others have worked to advance this agenda substantially, coordinating the sharing of preclinical compound libraries and clinical research.^85^^,^^86^ Another example of such work is the Accelerating COVID-19 Therapeutic Interventions and Vaccines or ACTIV public–private consortium, involving U.S. government agencies, the European Medicines Agency, and private industry.^87^ Initial identification of known candidate agents for treatment of COVID-19 has mostly been achieved with computer modeling, or in silico testing.^88^ Notable achievements on this front include protein interaction mapping,^89^ which yielded both clinical and preclinical candidate agents as well as the screening of agents with established human safety profiles such as the ReFRAME library. This catalog of more than 12,000 compounds has already been used to identify candidates for treatment of other infectious diseases such as tuberculosis and cryptosporidiosis.^90^

Although screening of individual agents for COVID-19 has progressed rapidly, screening for combination therapy or “cocktails” has been less robust, particularly when excluding trials of combinations including HCQ. No single agent may be highly effective alone, but combinations of various agents to target multiple points in the viral life cycle or to prevent resistance may prove efficacious, as demonstrated in HIV and hepatitis C.^91^^–^^93^ A priori screening for candidate COVID-19 therapeutic combinations in silico followed by in vitro synergy studies could lead more rapidly to clinical trials of effective treatment of COVID-19.^94^^,^^95^ However, the complexities associated with manufacturing and administration of combination therapies should be considered, and those with potential for affordability and availability in LMICs should be prioritized.

Despite a large number of ongoing clinical trials worldwide, few are completed and only a small portion are adequately powered, randomized, double-blinded, or placebo-controlled. Trial endpoints vary, and there are a disproportionate number of trials for certain agents. The lack of patent rights for most of the identified agents may explain limited investment and coordination support from industry partners. Moreover, a small fraction of trials are currently being conducted in LMICs, which is important as outcomes in LMICs could differ due to genetic variability in strains and other host and health system factors specific to local contexts. Large clinical trials in LMICs often require highly collaborative international partnerships, and in-person site visits and collaboration have been curtailed during the pandemic. Also, diagnostic resources and capacity are more limited in LMICs, posing a 3-fold challenge: impeding modeling of the spread of the pandemic to facilitate well-planned trials, reducing identification and recruitment of proven cases, and further limiting diagnostic capacity for direct research use.

**Figure 2.**
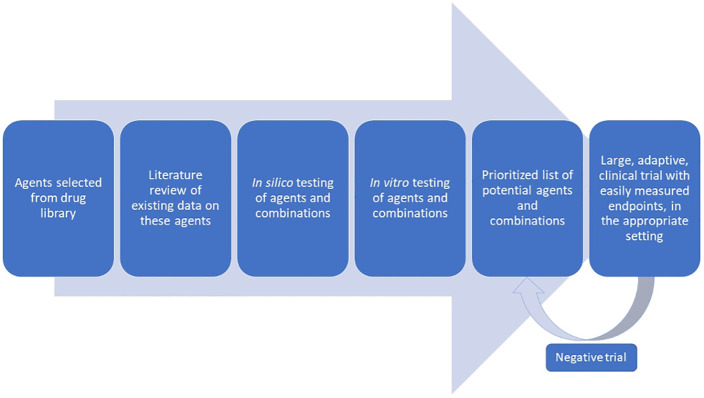
Proposed workflow for identifying effective COVID-19 therapeutics. This figure appears in color at www.ajtmh.org.

Although these challenges are significant, they are not insurmountable. New drug candidates appear rapidly, and a coordinated, adaptive trial design should be used. Focused clinical endpoints that do not require extensive resources can provide rigor without burdening researchers with added cost and complexity. Designing such trials requires broad expertise and thus broad collaboration. Such trial protocols could then be distributed, decreasing burdens on aspiring trialists and increasing sample sizes and sites to improve the power and generalizability of findings. Bearing these principles in mind and adapting from other proposed frameworks,^96^ we propose a drug development framework with an intentional focus on the unmet needs in LMICs (Figure 2). Indeed, some or all of the approaches discussed here have been used by groups such as RECOVERY, SOLIDARITY, and ACTT1-4, to name a few. An example of work to coordinate such large efforts with open collaboration is the COVID-19 Clinical Research Coalition, which has called for and facilitated large RCTs in LMICs and has made research materials available for these studies.^97^ Many of the agents studied, such as HCQ, are of lower interest given repeated negative findings. However, this framework of collaboration, in combination with the screening techniques discussed here, and greater political and financial commitment, could lead more rapidly to actionable results and much-needed therapies.

## CONCLUSIONS

Expensive and injectable tools against COVID-19—whether vaccines or therapeutics such as remdesivir and monoclonal antibodies that are not included in this review—present challenges in production, distribution, and uptake in LMICs. Agents alone or in combination that are accessible, affordable, and easily administered should be emphasized in pragmatic, adaptive clinical trials specifically targeted for LMICs. Such efforts could yield not only more rapid results but also a truly worldwide impact whereby we can prevent the COVID-19 pandemic from exacerbating global health inequities.

## Supplemental appendix


Supplemental materials
