# Supplementary material for: COVID-19 Therapeutics for Low- and Middle-Income Countries: A Review of Candidate Agents with Potential for Near-Term Use and Impact
Source: Am J Trop Med Hyg. 2021 Jul 16;105(3):584–95. doi: 10.4269/ajtmh.21-0200 (PMC8592342; doi:10.4269/ajtmh.21-0200)
Supplement: Supplementary file 1 [file tpmd210200.SD1.docx]

**Supplemental Appendix.** Additional agents that did not meet final search criteria but were identified in prior search that included trials registered before January 14, 2021 and had at least 3 registered trials.

***Bemcentinib***

Axl is one of a group of tyrosine kinase receptors which has previously been shown to mediate the entry of Zika virus^98^. More recently, an *in silico* screening of approximately 9000 United States Food and Drug Administration (FDA) approved drugs for their potential to interfere with the ability of SARS-CoV-2 to evade innate immune recognition identified the Axl kinase inhibitor bemcentinib as a candidate^99^. Moreover, bemcentinib may prevent downregulation of the innate immune response, including IFN production. Lastly, it may inhibit viral cell entry as with Zika, West Nile, and Ebola viruses. Bemcentinib has been included in the United Kingdom’s ACcelerating COVID-19 dRUG Development (ACCORD) trial and robust data are anticipated.

***Doxycycline***

Doxycycline is another repurposed drug with potential to treat COVID-19 given its antiviral and anti-inflammatory properties. It has showed in vitro activity against SARS-CoV2 infected Vero E cells with median effective concentration EC50 comparable with oral and parenteral formulation^100^ and exhibits antiviral activity against several RNA viruses like dengue virus. The antiviral effect mechanism may be due to upregulation of the intracellular zinc finger antiviral protein which in turns binds to specific viral mRNAs and represses RNA translation, an effect previously demonstrated with Ebola, HIV, Zika and Influenza A viruses^101, 102^. Doxycycline also stimulates pro-inflammatory cytokines including interleukin-6 and tumor necrosis factor-alpha^103^ and could inhibit SARS CoV2 papain-like protease ^104, 105^. The PRINCIPLE trial includes an arm for usual care plus doxycycline in the outpatient setting. In another small scale trial, ivermectin/doxycycline combination vs standard of care was shown to reduce time to recovery and disease progression^106^.

***Fingolimod and opaganib***

Acute lung injury and pulmonary edema which have been extensively described in severe COVID-19 patients are characterized by an increased in vascular permeability. S1P was proven to be a potent angiogenic factor that enhances lung cell wall integrity and an inhibitor of vascular permeability and alveolar flooding in pre-clinical models of acute lung injury^107^. As an S1P analogue, it has been postulated the oral Fingolimod, which is used to treat multiple sclerosis, might also decrease COVID-19-associated increased vascular permeability and associated lung injury^108^. One phase 2 trial was planned to investigate the role of Fingolimod in COVID-19 patients but was later withdrawn. Opaganib is another oral agent that modulates decreases levels of S1P. Although no in-vitro evaluation of antiviral activity of opaganib against the SARS CoV2 virus have been published, there has been previous publications demonstrating antiviral effects of targeting the SphK2 enzyme against the Chikungunya virus^109^, Influenza viruses^110^. Opaganib was used on the basis of compassionate use in Israel in 7 patients with reported improvement in both clinical and laboratory parameters^111^. Two trials have since then assessed the role of Opaganib. A phase 2 trial enrolled 40 patients in the US, but was not powered for statistical significance and was focused on efficacy defined by reduction in total oxygen requirement over the course of treatment up to 14 days (ClinicalTrials.gov NCT04414618). Another global phase 2/3 trial of Opaganib in patients with severe COVID-19 pneumonia (ClinicalTrials.gov NCT04467840) is currently enrolling patients. Another point to note is the unclear effect of Fingolimod-induced lymphopenia would have on COVID-19 patients given that lymphopenia is associated with worse outcomes in COVID-19 patients^112^.

***Maraviroc***

Computational bioinformatics have identified chemokine receptor antagonist maraviroc (MRV) as a potential antagonist to the SARS C0V2 main protease M^pro^, 3CL ^pro^, the function of which is regulating replication and transcription by cleaving polyproteins to generate non-structural proteins (NSPs). From a replicase-transcriptase complex. Maraviroc was found to bind to the substrate-binding pocket of M^pro^ forming a significant number of non-covalent interactions^113^. This has not been substantiated with in vitro studies as MRV had EC_50_ values above 40 µM (compared with Remdesevir EC_50_ of 1.1)^114^ thus making MRV an unattractive agent clinically. Nevertheless given the pressing needs created by the pandemic, 3 trials were initiated to investigate the potential benefit of maraviroc for the treatment of COVID-19 patients: 2 are currently recruiting in Barcelona Spain^97^ and Rhode Island Providence Hospital USA^115^. A third trial in Mexico City has maraviroc alone or maraviroc in addition to favipiravir as treatment arms is not yet recruiting.

***Umifenovir***

Originally licensed in Russia in 1993 for treatment and prophylaxis of Influenza, umifenovir was found to inhibit SARS-CoV reproduction *in vitro* and clinical trials were started. A retrospective review of 81 hospitalized, non-intensive care unit patients with COVID-19 in China showed no significant differences in outcomes other than longer hospital stays in the umifenovir group^116^. More recently, a meta-analysis of 12 studies, both retrospective and prospective, including a total of 1052 patients, found only a slightly higher rate of PCR-negative testing on hospital day 14 (CI: 1.04 to 1.55)^117^. Given that the end points of the included studies were predominantly symptomatic and laboratory-based with some composite end point, no mortality comparison was made.

**Appendix References**

98. Meertens L, Labeau A, Dejarnac O, Cipriani S, Sinigaglia L, Bonnet-Madin L, Le Charpentier T, Hafirassou ML, Zamborlini A, Cao-Lormeau V-M, Coulpier M, Missé D, Jouvenet N, Tabibiazar R, Gressens P, Schwartz O, Amara A, 2017. Axl Mediates ZIKA Virus Entry in Human Glial Cells and Modulates Innate Immune Responses. Cell Reports 18: 324-333.

99. Encinar JA, Menendez JA, 2020. Potential Drugs Targeting Early Innate Immune Evasion of SARS-Coronavirus 2 via 2’-O-Methylation of Viral RNA. Viruses 12: 525.

100. Gendrot M, Andreani J, Jardot P, Hutter S, Boxberger M, Mosnier J, Le Bideau M, Duflot I, Fonta I, Rolland C, In vitro antiviral activity of doxycycline against SARS-CoV-2.

101. Cai J, Wang X, Zhao B, Yao W, Wang X, Zhu Q, Zeng M, 2014. Prevalence, genetic drift of haemagglutinin, and antiviral resistance of influenza A/H3N2 viruses circulating in Shanghai in children during 2009-2012. J Med Virol 86: 1026-33.

102. Rothan HA, Mohamed Z, Paydar M, Abd Rahman N, Yusof R, 2014. Inhibitory effect of doxycycline against dengue virus replication in vitro. Archives of virology 159: 711-718.

103. Fredeking TM, Zavala-Castro JE, González-Martínez P, Moguel-Rodríguez W, C Sanchez E, J Foster M, A Diaz-Quijano F, 2015. Dengue patients treated with doxycycline showed lower mortality associated to a reduction in IL-6 and TNF levels. Recent patents on anti-infective drug discovery 10: 51-58.

104. Barretto N, Jukneliene D, Ratia K, Chen Z, Mesecar AD, Baker SC, 2005. The papain-like protease of severe acute respiratory syndrome coronavirus has deubiquitinating activity. Journal of virology 79: 15189-15198.

105. Cai J, Liu W, Wong CW, Zhu W, Lin Y, Hu J, Xu W, Zhang J, Sander M, Wang Z, Dan J, Zhang J, Liu Y, Guo L, Qin Z, Liu X, Liu Y, Yan G, Wu S, Liang J, 2020. Zinc-finger antiviral protein acts as a tumor suppressor in colorectal cancer. Oncogene 39: 5995-6008.

106. Hashim HA, Maulood MF, Rasheed AM, Fatak DF, Kabah KK, Abdulamir AS, 2020. Controlled randomized clinical trial on using Ivermectin with Doxycycline for treating COVID-19 patients in Baghdad, Iraq. medRxiv.

107. Natarajan V, Dudek SM, Jacobson JR, Moreno-Vinasco L, Huang LS, Abassi T, Mathew B, Zhao Y, Wang L, Bittman R, 2013. Sphingosine-1–phosphate, FTY720, and Sphingosine-1–phosphate receptors in the pathobiology of acute lung injury. American journal of respiratory cell and molecular biology 49: 6-17.

108. Lisi L, Lacal PM, Barbaccia ML, Graziani G, 2020. Approaching coronavirus disease 2019: Mechanisms of action of repurposed drugs with potential activity against SARS-CoV-2. Biochemical pharmacology: 114169.

109. Reid SP, Tritsch SR, Kota K, Chiang C-Y, Dong L, Kenny T, Brueggemann EE, Ward MD, Cazares LH, Bavari S, 2015. Sphingosine kinase 2 is a chikungunya virus host factor co-localized with the viral replication complex. Emerging microbes & infections 4: 1-9.

110. Cai MJ, Zhan FX, Kong XN, Zhu SZ, Cui Y, Wang Q, 2018. RING domain of zinc finger protein like 1 is essential for cell proliferation in endometrial cancer cell line RL95-2. Gene 677: 17-23.

111. Kurd R, Ben-Chetrit E, Karameh H, Bar-Meir M, 2020. Compassionate Use of Opaganib For Patients with Severe COVID-19. medRxiv.

112. Wynants L, Van Calster B, Collins GS, Riley RD, Heinze G, Schuit E, Bonten MM, Dahly DL, Damen JA, Debray TP, 2020. Prediction models for diagnosis and prognosis of covid-19: systematic review and critical appraisal. bmj 369.

113. Shamsi A, Mohammad T, Anwar S, AlAjmi MF, Hussain A, Rehman M, Islam A, Hassan M, 2020. Glecaprevir and Maraviroc are high-affinity inhibitors of SARS-CoV-2 main protease: possible implication in COVID-19 therapy. Bioscience Reports 40.

114. Okamoto M, Toyama M, Baba M, 2020. The chemokine receptor antagonist cenicriviroc inhibits the replication of SARS-CoV-2 in vitro. Antiviral Research 182: 104902.

115. NLM COVID-19 Interventional Trials. Available at: https://clinicaltrials.gov/ct2/results?cond=COVID-19&age_v=&gndr=&type=Intr&rslt=&Search=Apply. Accessed 22 Oct, 2020.

116. Lian N, Xie H, Lin S, Huang J, Zhao J, Lin Q, 2020. Umifenovir treatment is not associated with improved outcomes in patients with coronavirus disease 2019: a retrospective study. Clinical Microbiology and Infection 26: 917-921.

117. Huang D, Yu H, Wang T, Yang H, Yao R, Liang Z, 2020. Efficacy and safety of umifenovir for coronavirus disease 2019 (COVID‐19): A systematic review and meta‐analysis. Journal of Medical Virology.
